# Assessment of High-Power Catheter Ablation in Patients With Atrial Fibrillation: A Meta-Analysis

**DOI:** 10.3389/fcvm.2021.609590

**Published:** 2021-10-20

**Authors:** Zhi-Jie Mao, Yan Pei, Hui Lin, Yin Xiang, Zhou-Qing Huang, Fang-Yi Xiao, Yi-He Chen

**Affiliations:** ^1^Department of Cardiology, The First Affiliated Hospital of Wenzhou Medical University, Wenzhou, China; ^2^Department of Cardiology, The First People's Hospital of JinZhong, Yuci, China; ^3^Department of Respiratory, The Second Affiliated Hospital and Yuying Children's Hospital of Wenzhou Medical University, Wenzhou, China; ^4^Department of Cardiology, Xinhua Hospital Affiliated to The Medical School of Shanghai Jiaotong University, Shanghai, China

**Keywords:** high-power ablation, ablation lesion, atrial fibrillation, catheter ablation, meta-analysis

## Abstract

**Background:** High-power radiofrequency (RF) catheter ablation was considered as a promising alternative strategy to conventional-power ablation in the treatment of patients with atrial fibrillation (AF). This study sought to compare the efficacy and safety of high-power energy delivery to that of conventional-power setting in AF catheter ablation.

**Methods:** We performed a systematic review of relevant literature in Pubmed, Embase, Cochrane library, and Google Scholar database. Sixteen eligible studies totaling 3,307 patients (1,929 for high-power ablation; 1,378 for conventional-power ablation) met inclusion criteria.

**Results:** During a median 12 month follow-up, high-power ablation showed a significantly higher AF/atrial tachycardia-free survival rate in comparison with conventional-power ablation (risk ratio [RR] 1.09, 95% CI 1.02 to 1.15, *p* = 0.008). Notably, a high-power strategy convincingly decreased the procedure time (weighted mean difference [WMD] −46.11 min, 95% CI −59.15 to −33.07, *p* < 0.001) and RF ablation time (WMD −19.19 min, 95% CI −24.47 to −13.90, *p* < 0.001), along with reduced fluoroscopy time (WMD −7.82 min, 95% CI −15.13 to −0.68, *p* = 0.036). In addition, there was no perceptible difference in the potential risk of procedure-related complications between these two approaches (RR 0.81, 95% CI 0.48 to 1.37, *p* = 0.428).

**Conclusions:** High-power RF catheter ablation was associated with an improvement in long-term sinus rhythm maintenance for treatment of AF, without exacerbating the risk of adverse events during the procedure. Impressively, high-power pulmonary vein isolation had the potential to shorten the application duration and minimize fluoroscopic exposure.

## Introduction

Radiofrequency (RF) catheter ablation is an effective option for patients with symptomatic, drug-refractory atrial fibrillation (AF) (1). Nevertheless, durable pulmonary vein isolation (PVI) is still a clinical challenge in achieving long-term atrial arrhythmia-free survival. Emerging evidence indicates that contact force, catheter stability, and particularly RF energy settings are the main determinants of irreversible, continuous lesion creation (2, 3). Currently, PVI is commonly applied with a low power output (20–35 W) for a relatively long duration (4, 5). However, the rate of pulmonary vein reconduction remains substantial and subsequently increases the risk for recurrence.

In view of this, a high-power ablation strategy is increasingly employed to treatment of AF in order to generate more effective lesions while shortening the duration of energy delivery. Mechanistically, high-power ablation is linked with a wider, continuous tissue injury and composed of lower depth (6, 7). Remarkably, recent studies show an impressive efficacy of high-power ablation in maintenance of sinus rhythm without an increased risk for extracardiac damage in clinical setting (8–10). Compared to variation of radiofrequency output (ranged from 40 to 90 W), relatively few patients and potential selection bias introduced by observational data may collectively limit the strength and reliance of evidence.

Therefore, we conduct a comprehensive meta-analysis of available clinical studies to compare the efficacy and safety of high-power ablation with conventional-power ablation in patients undergoing treatment for AF.

## Methods

The study was registered with PROSPERO (CRD42020160991) and reported in accordance with the PRISMA statement.

### Literature Search

We systematically searched the electronic databases of Pubmed, Embase, Cochrane library, and Google Scholar from inception through June 2021 to identify the trials comparing radiofrequency catheter ablation with high power vs. conventional power for patients with atrial fibrillation. No language restrictions were set. The following keywords were used for search: “atrial fibrillation” or “AF,” AND “high power” or “higher power” or “high output' or “higher output.” Additional articles were searched through review of reference lists of related original and review articles which cited eligible manuscripts.

### Eligibility Criteria and Data Extraction

Studies that fulfilled the following criteria were eligible for further analysis: (i) observational studies or randomized control trials (RCTs); (ii) high-power RF ablation vs. conventional-power RF ablation; and (iii) provided the AF/atrial tachycardia relapse during a minimum of 6 month follow-up. A high-power RF ablation strategy was set as energy ≥40 W during the procedure. AF/atrial tachycardia relapse was defined by any documented episode of AF, atrial flutter, or atrial tachycardia lasting >30 s after the blanking period. Studies that enrolled patients with an initially failed atrial ablation or previous cardiac surgery were excluded. Subsequently, two investigators (Zhi-Jie Mao and Yi-He Chen) screened the included studies and extracted the following data: author name, year of publication, study design, AF type, follow-up duration, clinical monitoring during follow-up, ablation protocol, and technical details. Discrepancies that were unable to be resolved were discussed by an additional investigator (Hui Lin). Baseline characteristics of the study population (i.e., mean age, gender, sample size, left atrial diameter, CHA_2_DS_2_-VASc score, left ventricular ejection fraction [LVEF], body mass index [BMI], and comorbidities) were also extracted. The primary endpoint of efficacy was AF/atrial tachycardia-free survival, while the primary endpoints of safety were procedure time, RF ablation time, fluoroscopy time, or procedure-related complications (e.g., esophageal fistula, tamponade or pericarditis, stroke or transient ischemic attack [TIA], vascular-related adverse events, phrenic nerve palsy, pulmonary vein [PV] stenosis, pulmonary edema, cardiovascular ischemic attack, and even death).

### Statistical Analysis

Statistical analysis was conducted using STATA version 12.0 (StataCorp, College Station, TX, USA) and SPSS version 20.0 (SPSS Inc., IL). Categorical variables (AF/atrial tachycardia-free survival and procedure-related complications) and continuous variables (procedure time, ablation time, and fluoroscopy time) were presented as risk ratios (RRs) or weighted mean difference (WMDs) with corresponding 95% confidence intervals (CIs), respectively. Random-effect models were used according to the DerSimonian & Laird method. Heterogeneity among studies was estimated by Q test and quantified with *I*^2^ statistic. Publication bias for the pooled estimates was visually assessed by funnel plot and supplemented with Egger's test. Sensitivity analysis was performed by excluding each study for efficacy outcomes of AF/atrial tachycardia-free survival. To explore the impact of potential effect modifiers on outcomes and the possible sources of heterogeneity, subgroup analysis was conducted according to the following subsets: use of contact force (CF)-sensing catheter or ablation index (AI) guidance. A two-tailed *p* < 0.05 was considered as the threshold for statistical significance.

## Results

**Figure 1:**
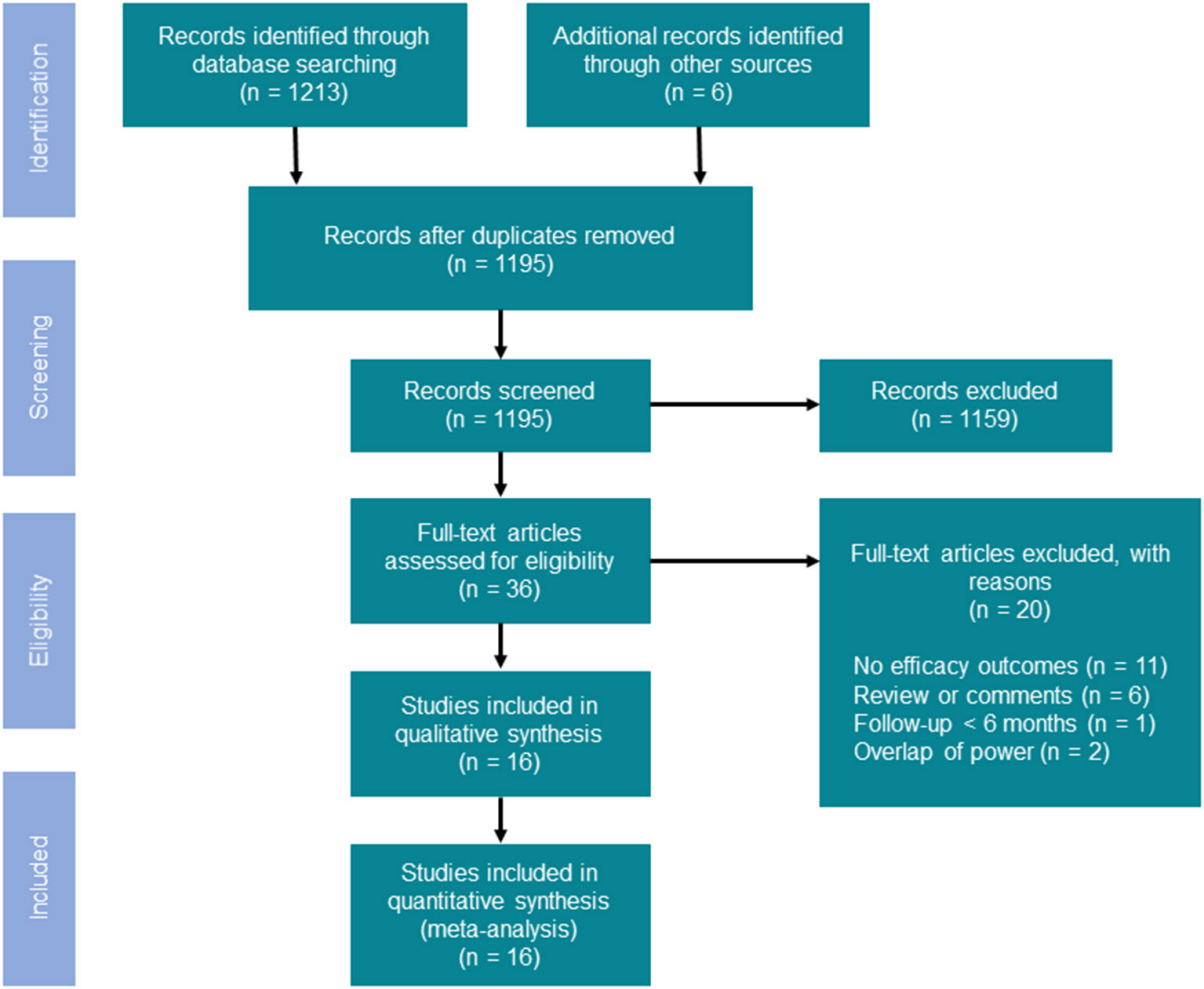
Flowchart of the search strategy.

The initial database search yielded a total of 1,195 articles. Of these, 36 potentially relevant articles were retained for full-text review after screening titles and abstracts. Sixteen studies comprising 3,307 patients (1,929 assigned to high-power group and 1,378 assigned to conventional-power group) met eligibility criteria for further meta-analysis (Figure 1). Details about the included studies and patient demographics are summarized in Tables 1–3. Of these studies, only two was RCT and the remaining followed either prospective (*n* = 8) and retrospective (*n* = 6) design. The majority of studies were executed in North America and Europe from 2006 to 2021. The mean age was 61.9 years. The proportion of male patients ranged from 60.4 to 84.3%. The median follow-up duration was 12 months. In the high-power group, RF application was mainly set as ≥40 W for the encirclement. Over half of included studies utilized CF-sensing catheter during the ablation. Meanwhile, additional AI guidance was conducted in the studies of Okamatsu et al. (22), Shin et al. (24), Kyriakopoulou et al. (19), Ejima et al. (20), and Hansom et al. (25). There was no significant difference in baseline patient characteristics between these two approaches (Table 3).

**Table 1:** Main characteristics of included studies.

**Author**	**State**	**Design**	**Patients**		**AF type**	**Age**	**Male (%)**	**Ablation protocol**		**Catheter parameters**	**Follow-up (months)**
			**HP**	**CP**				**HP**	**CP**		
Baher et al. (11)	USA	OS, R	574	113	ParAF, PerAF	68.9	78.7	50 W, 5 s	25–35 W, 10–30 s	3.5-mm irrigated tip, CF	30
Pambrun et al. (12)	France	OS, P	50	50	ParAF	63.8	65.0	40–50 W	25–30 W	3.5-mm irrigated tip, CF	12
Nilsson et al. (13)	Denmark	OS, R	45	45	ParAF, PerAF	53.0	73.3	45 W, 20 s	30 W, 120 s	5-mm irrigated tip	15
Kanj et al. (14)	USA	RCT	61	60	ParAF, PerAF	60.5	82.6	50 W	35 W	3.5-mm irrigated tip	6
Matiello et al. (15)	Spain	OS, P	89	42	ParAF, PerAF	53.3	73.3	40 W	30 W	5-mm irrigated tip	12
Yamada et al. (16)	Japan	OS, R	61	47	ParAF	57.0	84.3	40 W, 60 s	30 W, 60 s	4-mm or 8-mm tip	6
Vassallo et al. (9)	Brazil	OS, R	41	35	ParAF, PerAF	62.6	73.6	45–50 W, 6 s	30 W, 30 s	3.5-mm irrigated tip, CF	12
Bunch et al. (17)	USA	OS, P	402	402	ParAF, PerAF	66.8	64.1	50 W, 2–15 s	30 W, 5–20 s	3.5-mm irrigated tip, CF	12
Kottmaier et al. (18)	Germany	OS, P	97	100	ParAF	60.8	59.4	70 W, 5–7 s	30–40 W, 20–40 s	4-mm irrigated tip	12
Kyriakopoulou et al. (19)	Belgium	OS, R	80	105	ParAF	65.3	60.4	40 W	35 W	3.5-mm irrigated tip, CF, AI	12
Ejima et al. (20)	Japan	OS, P	60	60	ParAF	64.9	71.7	50 W	25–40 W	3.5-mm irrigated tip, CF, AI	12
Yazaki et al. (21)	Japan	OS, R	32	32	ParAF, PerAF	63.5	73.4	50 W, 8–12 s	25–40 W, 15–40 s	3.5-mm irrigated tip, CF	10
Okamatsu et al. (22)	Japan	OS, P	20	20	ParAF, PerAF	66.5	70.0	40–50 W	20–30 W	3.5-mm irrigated tip, CF, AI	6
Yavin et al. (23)	USA	OS, P	112	112	ParAF, PerAF	63.6	67.0	45–50 W, 8–15 s	20–35 W, 20–30 s	3.5-mm irrigated tip, CF	19
Shin et al. (24)	Korea	RCT	100	50	ParAF, PerAF	58.2	76.0	40–50 W, 10–20 s	30 W, 40 s	3.5-mm irrigated tip, CF, AI	12
Hansom et al. (25)	Canada	OS, P	107	107	ParAF, PerAF	62.0	70.1	50 W, 6–10 s	20–35 W, 20–40 s	3.5-mm irrigated tip, CF, AI	12

*HP, high-power ablation; CP, conventional-power ablation; AF, atrial fibrillation; OS, observational study; RCT, randomized control trail; P, prospective; R, retrospective; ParAF, paroxysmal atrial fibrillation; PerAF, persistent atrial fibrillation; W, watt; CF, contact force-sensing catheter; AI, ablation index*.

**Table 2:** Detailed clinical follow-up information of included studies.

**Author**	**Clinical monitoring during follow-up**	**Blanking period**	**Definition of recurrence**
Baher et al. (11)	30 or 60 d event monitors after ablation and at the 3 month follow-up. Patients were followed at 3-, 6-, 12 month intervals. Additional home monitoring was ordered in case of AF symptoms	3 months	≥30-s AF or flutter
Pambrun et al. (12)	10 d Holter monitoring at 1, 3, 12 months, 24 h Holter monitoring at 6 and 9 months. Additional 24 h Holter was obtained in case of AF symptoms	NA	>30-s AF or atrial tachycardia
Nilsson et al. (13)	NA	1 month	Atrial arrhythmias
Kanj et al. (14)	Arrhythmia transmitter was used to monitor events during the first 6 months	2 months	Atrial arrhythmias
Matiello et al. (15)	24 h Holter monitoring at 1, 4, and 7 months, and every 6 months thereafter. ECG was performed in case of arrhythmia symptoms	3 months	AF or left atrial flutter
Yamada et al. (16)	24 h Holter monitoring at 2 weeks, 1 month, and every month thereafter	NA	Atrial arrhythmias
Vassallo et al. (9)	ECG at 3, 6, 9, and 12 months and 24 h Holter monitoring at 6 and 12 months	3 months	Atrial arrhythmias
Bunch et al. (17)	Ambulatory monitoring at 3 month intervals post ablation up to the first year	3 months	AF or atrial flutter
Kottmaier et al. (18)	7 d Holter monitoring at 3, 6, and 12 months	6 weeks	AF or atrial tachycardia
Kyriakopoulou et al. (19)	Holter monitoring at 12 months	3 months	>30-s atrial tachyarrhythmias
Ejima et al. (20)	ECG and 24 h Holter monitoring at 3, 6, 9, and 12 months and every 6 months thereafter	2 months	>30-s atrial tachyarrhythmias
Yazaki et al. (21)	ECG and 24 h Holter monitoring at 3, 6, 9, and 12 months and every 6 months thereafter	2 months	>30-s atrial tachyarrhythmias
Okamatsu et al. (22)	ECG and 24 h Holter monitoring at 3, 6, 9, and 12 months	3 months	>30-s atrial tachyarrhythmias
Yavin et al. (23)	14 d continuous or patient-triggered Holter monitoring	4 weeks	>30-s AF or atrial tachycardia
Shin et al. (24)	ECG and 24 h Holter monitoring at 3, 6, and 12 months. Additional ECG was performed in case of symptoms	3 months	>30-s AF, atrial tachycardia or atrial flutter
Hansom et al. (25)	ECG and 14 day Holter monitoring at 3, 6, and 12 months	3 months	>30-s atrial arrhythmias

*AF, atrial fibrillation; ECG, electrocardiogram; d, day; h, hour; NA, none available*.

**Table 3:** Baseline population demographics of included patients.

	**No. of studies**	**HP**	**CP**	***p*-value**
Age	16	61.9 ± 4.4	62.0 ± 5.0	0.917
Male (%)	16	71.7 ± 9.8	69.3 ± 8.2	0.471
Hypertension	12	53.1 ± 19.7	50.4 ± 18.8	0.733
Diabetes mellitus	9	20.1 ± 12.1	15.2 ± 8.6	0.333
Stroke/TIA	8	9.0 ± 3.7	8.9 ± 2.5	0.932
CHA_2_DS_2_-VASC	9	2.0 ± 0.4	2.1 ± 0.3	0.818
LA diameter	9	41.3 ± 2.7	41.0 ± 3.3	0.837
AF duration (year)	5	3.9 ± 2.0	4.3 ± 2.2	0.793
Heart failure	5	15.1 ± 19.1	14.8 ± 18.3	0.983
LVEF (%)	10	58.6 ± 4.2	58.6 ± 4.3	0.996
CAD	4	19.3 ± 6.7	16.9 ± 6.6	0.637
BMI	8	26.7 ± 2.4	26.8 ± 2.5	0.944

*HP, high-power ablation; CP, conventional-power ablation; TIA, transient ischemic attack; LA, left atrium; AF, atrial fibrillation; LVEF, left ventricular ejection fraction; CAD, coronary artery disease; BMI, body mass index*.

### Primary Efficacy Endpoints

**Figure 2:**
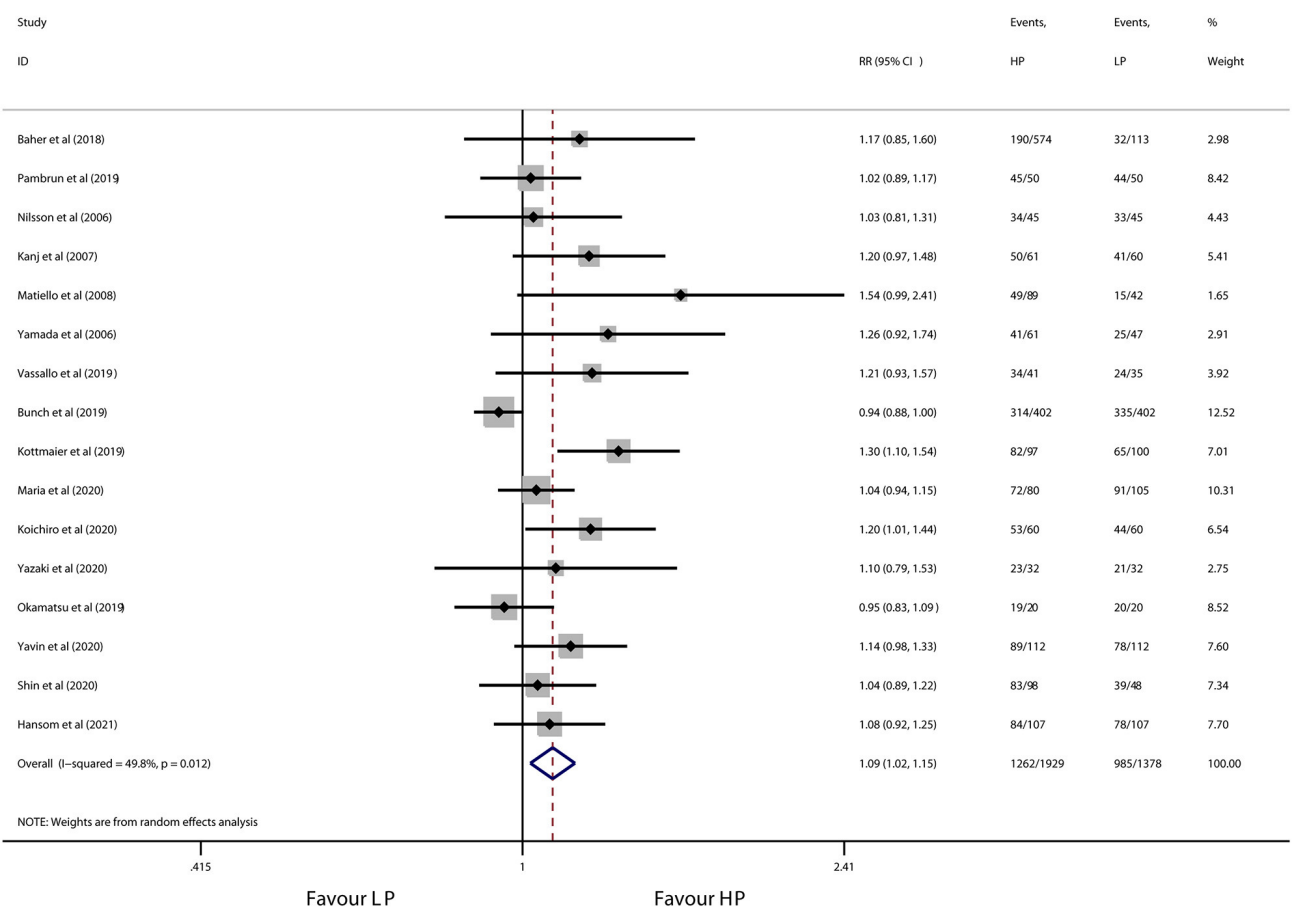
Forest plot of AF/atrial tachycardia-free survival for high-power ablation vs. conventional-power ablation.

All 16 studies contributed to the primary outcome of AF/atrial tachycardia-free survival. During a median follow-up of 12 months, high-power ablation was associated with a 9% relative increase in AF/atrial tachycardia-free survival as compared with conventional-power ablation (RR 1.09, 95% CI 1.02 to 1.15, *p* = 0.008) (Figure 2), with moderate heterogeneity across the studies (*I*^2^ = 49.8%, *p* = 0.012). Notwithstanding the publication bias detected by visual inspection of funnel plot and Egger's test (*p* = 0.001), the trim-and-fill test showed a similar result (adjust RR: 1.10, 95% CI 1.03 to 1.17). One study removed sensitivity analysis which revealed that individual data did not alter the results of this meta-analysis. Subgroup analyses according to use of CF-sensing catheter or AI-guided RF ablation are presented in Table 4. Of note, the pronounced benefit of high-power ablation over conventional-power ablation in maintenance of sinus rhythm was compromised by utilization of the CF-sensing catheter (RR 1.04, 95% CI 0.98–1.10, *p* = 0.164) or aided with AI guidance (RR 1.05, 95% CI 0.98–1.11, *p* = 0.142).

**Table 4:** Subgroup analysis of AF/atrial tachycardia-free survival.

**Subgroup**	**No. of studies**	**HP**		**CP**		**RR (95% CI)**	***p*-value**
		**Events**	**Patients**	**Events**	**Patients**		
CF-sensing catheter							
With	11	1,006	1,578	806	1,084	1.04 (0.98, 1.10)	0.164
Without	5	256	353	179	294	1.23 (1.10, 1.36)	<0.001
AI guided RF ablation							
With	6	334	397	293	372	1.05 (0.98, 1.11)	0.142
Without	10	928	1,532	692	1,006	1.13 (1.02, 1.25)	0.019
Total	16	1,262	1,929	958	1,378	1.09 (1.02, 1.15)	0.008

*HP, high-power ablation; CP, conventional-power ablation; CF, contact force; AI, ablation index; RF, radiofrequency; RR, risk ratios*.

### Primary Safety Endpoints

**Figure 3:**
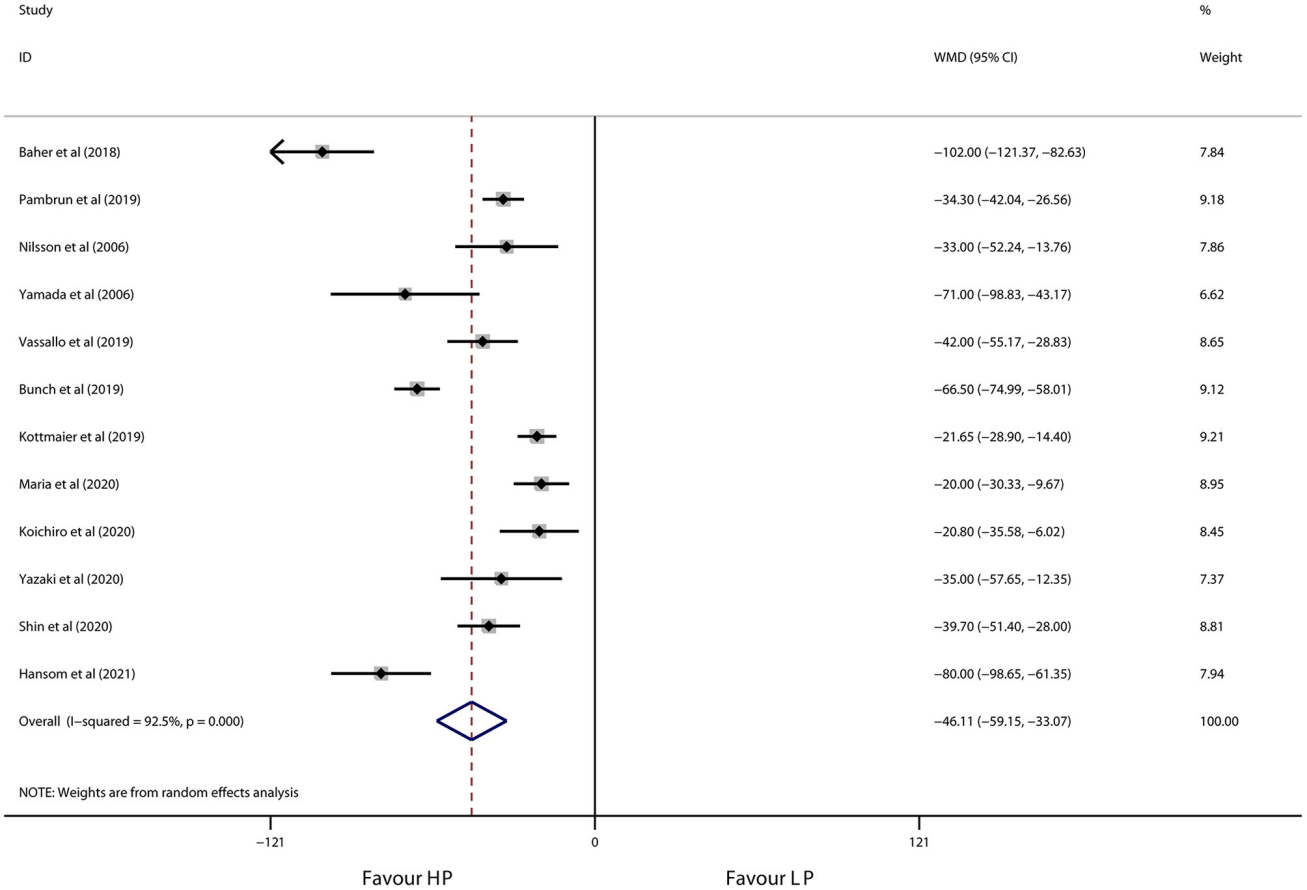
Forest plot of procedure time for high-power ablation vs. conventional-power ablation.

**Figure 4:**
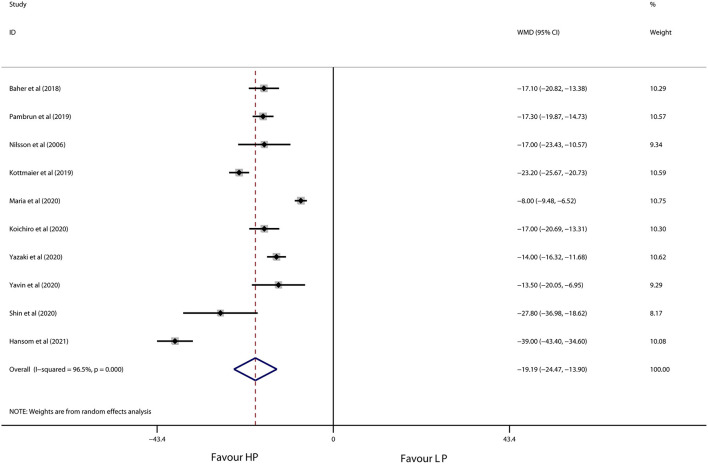
Forest plot of RF ablation time for high-power ablation vs. conventional-power ablation.

**Figure 5:**
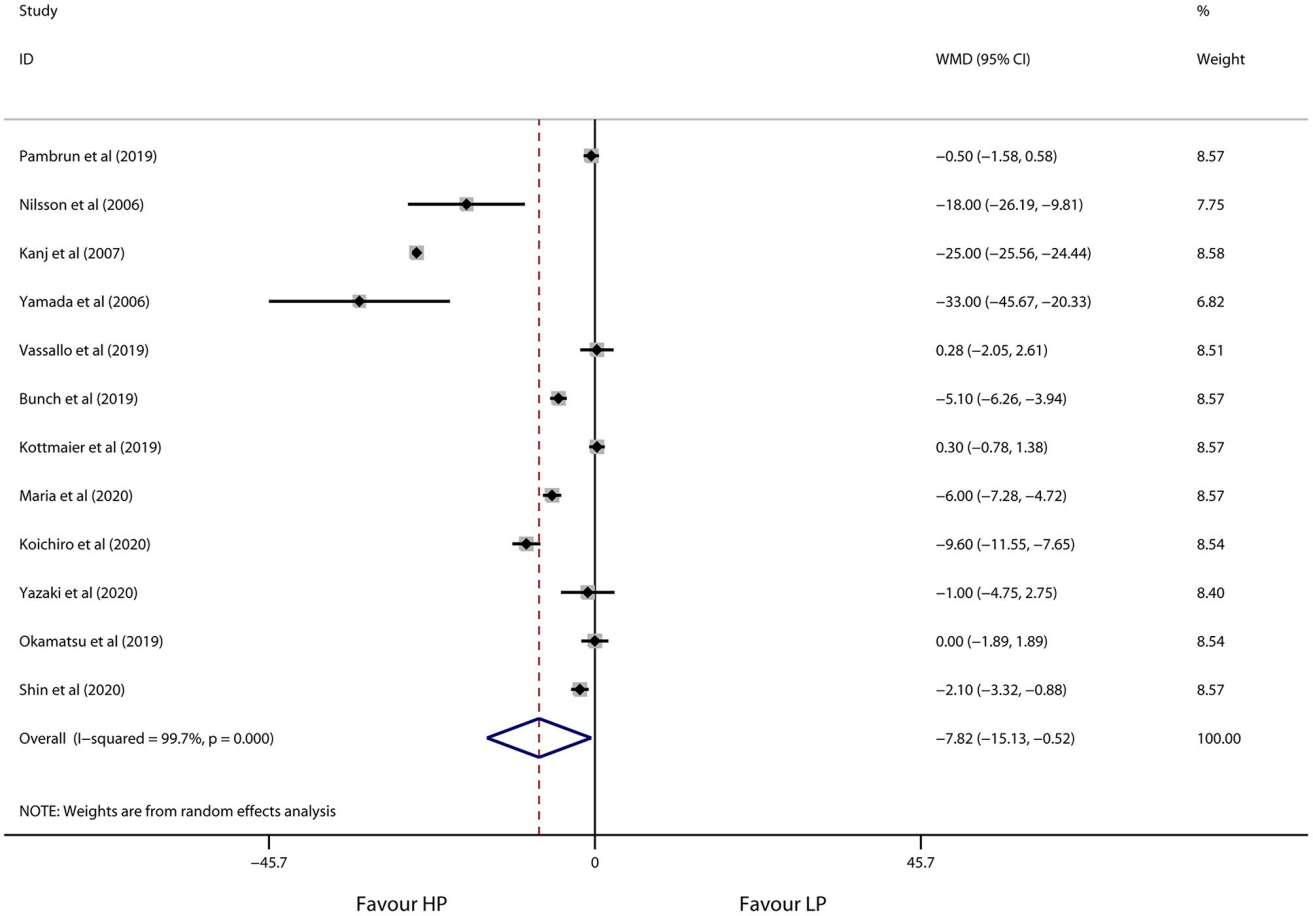
Forest plot of fluoroscopy time for high-power ablation vs. conventional-power ablation.

As shown in Figures 3, 4, high-power ablation was associated with a remarkable reduction in procedure time (WMD −46.11 min, 95% CI −59.15 to −33.07, *p* < 0.001) and RF ablation time (WMD −19.19 min, 95% CI −24.47 to −13.90, *p* < 0.001) compared to conventional-power ablation, with high heterogeneity for both (*I*^2^ = 83.2%, *p* = 0.003 and *I*^2^ = 96.5%, *p* < 0.001, respectively). Furthermore, high-power RF energy delivery also decreased fluoroscopy time in the high-power group (WMD −7.82 min, 95% CI −15.13 to −0.52, *p* = 0.036) in spite of the high heterogeneity (*I*^2^ = 99.8%, *p* < 0.001) (Figure 5). Systemic exclusion of each study did not change the pooled estimates and *p*-value. Accordingly, stratified by use of the CF-sensing catheter or AI-guided RF ablation also has no significant impact on these results (Table 5).

**Table 5:** Subgroup analysis of procedure time, RF ablation time, and fluoroscopy time.

**Subgroup**	**Procedure time**			**RF ablation time**			**Fluoroscopy time**		
	**No. of studies**	**WMD (95% CI)**	***p*-value**	**No. of studies**	**WMD (95% CI)**	***p*-value**	**No. of studies**	**WMD (95% CI)**	***p*-value**
**CF-sensing catheter**									
With	9	−48.29 (−63.72, −32.87)	<0.001	8	−18.92 (−24.90, −12.93)	<0.001	8	−3.08 (−5.31, −0.84)	0.007
Without	3	−38.70 (−63.13, −14.27)	0.002	2	−20.84 (−26.74, −14.94)	<0.001	4	−18.51 (−36.21, −0.81)	0.040
AI guided RF ablation									
With	5	−38.40 (−57.48, −19.33)	<0.001	5	−20.82 (−30.16, −11.47)	<0.001	5	−3.83 (−7.07, −0.60)	0.020
Without	7	−51.72 (−70.15, −33.28)	<0.001	5	−18.15 (−21.62, −14.69)	<0.001	7	−11.00 (−22.08, 0.07)	0.051
Total	12	−46.11 (−59.15, −33.07)	<0.001	10	−19.19 (−24.47, −19.30)	<0.001	12	−7.82 (−15.13, −0.52)	0.036

*CF, contact force; AI, ablation index; RF, radiofrequency; WMD, weighted mean difference; CI, confidence interval*.

**Figure 6:**
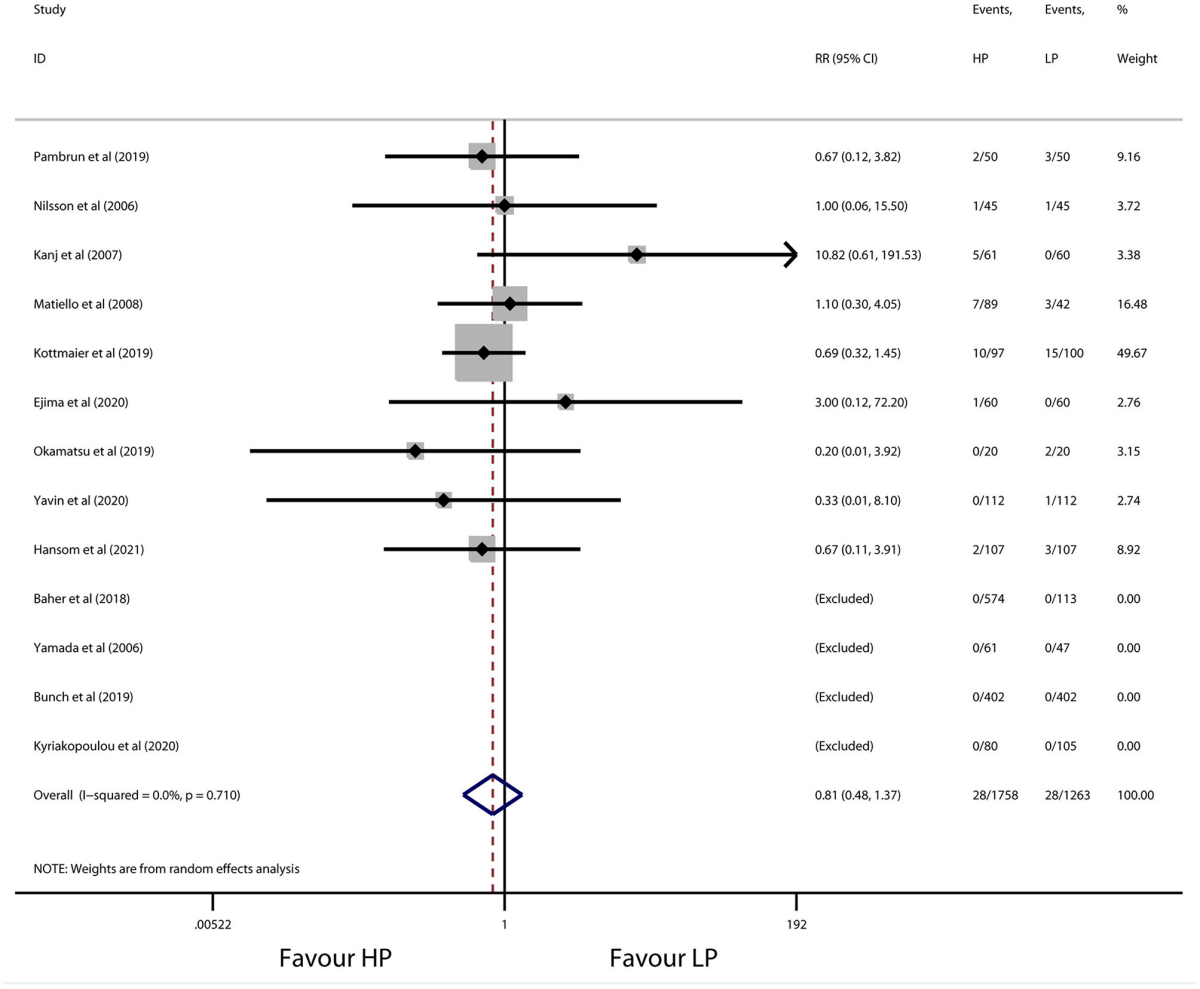
Forest plot of procedure-related complications for high-power ablation vs. conventional-power ablation.

The overall incidence of procedure-related complications was 1.59% (28 of 1,758 patients) in the high-power group and 2.22% (28 of 1,263 patients) in the conventional-power group. No significant difference was observed for patients with high-power ablation vs. conventional-power ablation (RR 0.81, 95% CI 0.48 to 1.37, *p* = 0.428) (Figure 6), with low heterogeneity (*I*^2^ = 0.0%, *p* = 0.710). Consistently, the results did not change after sensitivity and subgroup analysis (Table 6). Detailed information of complications during the procedure is presented in Table 7. No death, esophageal fistula, and PV stenosis occurred in either group. Notably, the predominant adverse events were vascular complications (e.g., groin hematoma, pseudoaneurysm, and arteriovenous fistula) with 0.74% patients in the high-power group and 1.74% patients in the conventional-power group. In addition, there was a similar prevalence of pericardial complications (0.29% vs. 0.24%), stroke/TIA (0.23% vs. 0.16%), and cardiovascular ischemic attack (0.11% vs. 0.08%) from both groups.

**Table 6:** Subgroup analysis of procedure-related complications.

**Subgroup**	**No. of studies**	**HP**		**CP**		**RR (95% CI)**	***p*-value**
		**Events**	**Patients**	**Events**	**Patients**		
**CF-sensing catheter**							
With	8	5	1,405	9	969	0.63 (0.23, 1.75)	0.374
Without	5	23	353	19	294	0.96 (0.45, 2.02)	0.907
**AI guided RF ablation**							
With	4	3	267	5	292	0.68 (0.17, 2.67)	0.586
Without	9	25	1,491	23	971	0.83 (0.47, 1.47)	0.528
Total	13	28	1,758	28	1,263	0.81 (0.48, 1.37)	0.428

*HP, high-power ablation; CP, conventional-power ablation; CF, contact force; AI, ablation index; RF, radiofrequency; RR, risk ratios*.

**Table 7:** Details of procedure-related complications.

**Complications**	**HP**			**CP**		
	**Events**	**Incidence (%)**	**Ratio (%)**	**Events**	**Incidence (%)**	**Ratio (%)**
Tamponade	2	0.11	7.1	2	0.16	7.1
Pericarditis	3	0.18	10.8	1	0.08	3.6
Stroke/TIA	4	0.23	14.3	2	0.16	7.1
Esophageal fistula	0	0.00	0.0	0	0.00	0.0
Vascular	13	0.74	46.5	22	1.74	78.6
PV stenosis (>50%)	0	0.00	0.0	0	0.00	0.0
Pulmonary edema	2	0.11	7.1	0	0.00	0.0
Cardiovascular ischemic attack	2	0.11	7.1	1	0.08	3.6
Death	0	0.00	0.0	0	0.00	0.0
PNP	2	0.11	7.1	0	0.00	0.0
Total	28	1.59	100	28	2.22	100

*HP, high-power ablation; CP, conventional-power ablation; TIA, transient ischemic attack; PV, pulmonary vein; PNP, phrenic nerve palsy*.

## Discussion

In the present analysis, high-power ablation was associated with an improved AF/atrial tachycardia-free survival rate during a 12 month median follow-up in comparison with conventional-power ablation. Importantly, high-power settings significantly reduced the procedure time and RF ablation time and alleviated fluoroscopy exposure. Of note, the incidence of procedure-related complications was comparable between these two approaches.

### Biophysical Mechanism of High-Power Ablation

RF catheter ablation transmits energy of alternating current with a frequency of 500–1,000 kHz, causing permanent tissue damage (26). Electrical currents through the tissue primarily generated resistive heating in the vicinity of the electrode tip, while deeper and extracardiac damage occurs as a consequence of passive heat conduction. Commonly, the ablation power setting was 20 to 30 W for a duration of 30 to 60 s in clinical practice (4, 5), whereas a longer duration of the current ablation strategy significantly extended the conductive heating phase and thus increased the risk of thermal injury to adjacent structures (i.e., lung hematoma/hemorrhage, phrenic nerve palsy, atrioesophageal fistula) (27, 28). Lesion formation was mainly based on the power and duration of RF application, which provided the rationale for high-power RF delivery with short duration by modifying the relationship between resistive and conductive heating (3). It was intended for rapidly generating lethal heating during the resistive phase and avoiding the distant tissue damage. Furthermore, higher-power and resultant shorter RF application could overcome the challenge of catheter dislodgement or inadequate catheter–tissue contact resulting in tissue edema, and improved the lesion efficacy. The mechanistic insight into the lesion biophysics metrics of higher-power ablation was initially evaluated in both *in vivo* and *ex vivo* models by Borne et al. (29). They found that 50 W/5 s was associated with a trend toward larger lesion diameter but a less lesion depth than 20 W/30 s. In addition, experimental data from Bourier et al. also reported a similar lesion geometry of high power with reduced lesion depth and extended lesion width in porcine thigh muscle preparations when compared with the standard approach (30). Subsequently, Leshem et al. further confirmed the efficacy and safety of high-power ablation in a beating heart swine model which resembled RF catheter ablation in clinical practice (31). Histopathologic examinations demonstrated an ~50% greater width (6.02 ± 0.2 mm vs. 4.43 ± 1.0 mm) and similar depth (3.58 ± 0.3 mm vs. 3.53 ± 0.6 mm) of atrial lesions vs. lower power. In terms of the thickness of the left atrium (0.5–3.5 mm), it was theoretically plausible that high-power ablation favored the creation of contiguous, transmural lesions which may facilitate the circumferential isolation of the pulmonary vein (32).

Intriguingly, a previous systematical review by Yuyun et al. reported a significant relationship between power output and primary effectiveness outcomes of AF catheter ablation and recommended the higher-power (>45-W), shorter-duration (15–20-s) strategy for RF ablation with optimized efficacy and safety profiles (33). Early in 2006, Yamada et al. retrospectively compared high-power output with conventional parameters in patients with paroxysmal AF (16). High-power ablation was associated with a lower AF recurrence (32% vs. 47%) during a short-term follow-up. In another randomized pilot trial by Kanj et al., higher-energy delivery presented a markedly greater efficacy for maintenance of sinus rhythm at the 6 month follow-up compared with the lower-power setting (14). Subsequently, Matiello et al. ensured the favorable clinical endpoints of long-term AF/atrial tachycardia-free survival in patients who underwent RF catheter ablation with high output (15). In view of this, the high-power RF ablation strategy was increasingly utilized for treatment of AF in recent studies. Chen et al. displayed a higher first-round PVI, lower acute reconnection of 50-W ablation, which translated into almost excellent clinical endpoints of sinus rhythm maintenance (96% patients) at 6 months (34). Accordingly, Kottmaier and colleagues, likewise, demonstrated that high-power ablation was associated with a significantly fewer atrial arrhythmia recurrence after 1 year (18). The present study reconfirmed the superiority of high-power ablation with a 10% increase in freedom from the AF rate during a median follow-up of 12 months. Therefore, the aforementioned results provided robust evidence for the feasibility and efficacy of high-power ablation in the treatment of AF.

Recently, Winkle et al. retrospectively examined the adverse events in 10,284 patients receiving AF ablation with a high-power setting (45–50 W/2–15 s) (35). This study exhibited an extremely low incidence of procedure-related complications. In the high-power group, only one atrio-esophageal fistula was found in the 11,436 ablations compared with 3 of the 2,538 ablations of the conventional-power group. In line with this, Baher et al. also detected identical esophageal thermal injury patterns between these two approaches assessed by late gadolinium enhancement MRI (11). Interestingly, data from the present work unmasked a relatively higher proportion of pericardial complication and stroke for high-power ablation despite comparable prevalence vs. conventional-power ablation. To our knowledge, steam pop and catheter char due to tissue overheating were regarded as the main culprit of tamponade and thrombus, which may be partially resolved by utilization of novel multielectrode catheters with a more sophisticated temperature feedback control system and advanced cooling techniques. Moreover, an optimal CF spectrum and/or AI-guided ablation may further minimize the collateral thermal injury during the high-power RF delivery.

Notably, high-power ablation constantly reduced the procedure time and RF ablation time and shortened the radiation exposure across the studies irrespective of supplementary CF or AI application. Elongation access to the systemic circulation may trigger thrombus formation and even uninterrupted anticoagulation. A prior study reported an ~13 to 20% frequency of subtle neurocognitive impairment after ablation of AF and disclosed a causal relationship between cognitive decline and left atrial access time (36). On this account, a high power accompanied with shorter duration may imperceptibly provide a guarantee of neuroprotection. Furthermore, this shortening also limited the excessive fluid load from catheter irrigation, which in turn minimized the risk of acute heart failure in patients with impaired cardiac function. Particularly, it was necessary to highlight that shorter ablation time could remarkably weaken the impact of heart beating and even deep breathing on catheter stability while ensuring irreversible atrial lesions and favorable long-term outcomes.

### Limitations

First, lack of individual patient-level data impeded the exploration of correlation between other effect modifiers (e.g., catheter, type of AF, left atrial dimension) and clinical outcomes. Second, the present results were largely driven by observational studies, which seemed more susceptible to potential biases. Third, there was a variation in the radiofrequency energy dosing of the high-power ablation strategy in different clinical centers (varied from 40 to 70 W), while in the conventional-power group, RF application was even set at 40 W from Kottmaier et al. and Ejima et al., which led to partial overlaps between these two approaches and may possibly dwarf the beneficial effect of the high-power setting (18, 20). Therefore, it was of great importance to standardize ablation parameters of high output strategy. Taking into account consistent safety outcomes in the included studies and the results of animal studies *in vivo*, >50 W may be an appropriate definition of “Real HPSD.” The benefits of strictly realistic HPSD may have been underestimated in this article. Finally, despite substantial heterogeneity among the studies, sensitivity and subgroup analyses demonstrated no difference in pooled results and provided robust evidence of its superiority in improving efficacy and safety outcomes.

## Conclusions

High-power ablation presents an incremental long-term efficacy in maintaining the sinus rhythm vs. conventional-power ablation in patients with AF. In addition, a high-power strategy significantly reduces the procedure burden and fluoroscopic exposure without increasing the risk of procedure-related complications.

## Data Availability Statement

The original contributions presented in the study are included in the article/supplementary material, further inquiries can be directed to the corresponding author/s.

## Author Contributions

F-YX provided the idea. Y-HC designed and subsequently guided this article and is responsible for the overall content as guarantor. HL and Z-QH assisted with the guiding and revising of the article. Other authors participated in writing and data processing statistics. All authors contributed to the article and approved the submitted version.

## Funding

This work was supported by the Natural Science Foundation of China (NSFC) (Grant No. 81900229) and Wenzhou Municipal Science and Technology Bureau (Grant No. Y20180079) to Y-HC. and the Natural Science Foundation of China (NSFC) (Grant No. 81900293) to Qian Wang.

## Conflict of Interest

The authors declare that the research was conducted in the absence of any commercial or financial relationships that could be construed as a potential conflict of interest.

## Publisher's Note

All claims expressed in this article are solely those of the authors and do not necessarily represent those of their affiliated organizations, or those of the publisher, the editors and the reviewers. Any product that may be evaluated in this article, or claim that may be made by its manufacturer, is not guaranteed or endorsed by the publisher.
